# Innovative nebulization delivery of lipid nanoparticle-encapsulated siRNA: a therapeutic advance for *Staphylococcus aureus*-induced pneumonia

**DOI:** 10.1186/s12967-024-05711-9

**Published:** 2024-10-15

**Authors:** Meiqi Meng, Yue Li, Jiachao Wang, Xiaonan Han, Xuan Wang, Hongru Li, Bai Xiang, Cuiqing Ma

**Affiliations:** 1 Department of Immunology, Key Laboratory of Immune Mechanism and Intervention On Serious Disease in Hebei Province, Hebei Medical University, Shijiazhuang, 050017 Hebei China; 2 Department of Pharmaceutics, School of Pharmaceutical Sciences, Hebei Medical University, Shijiazhuang, Hebei 050017 People’s Republic of China; 3 Department of Mathematics, Hebei Medical University, Shijiazhuang, 050017 Hebei China; 4 National Key Laboratory of New Pharmaceutical Preparations and Excipients, Shijiazhuang, 050035 People’s Republic of China; 5 Hebei Key Laboratory of Innovative Drug Research and Evaluation, Shijiazhuang, 050017 People’s Republic of China

**Keywords:** Lipid nanoparticle, Intratracheal nebulization, siRNA, Integrin α5β1, Anti-infection

## Abstract

**Background:**

Integrin α5β1 plays a crucial role in the invasion of nonphagocytic cells by *Staphylococcus aureus* (*S. aureus*), thereby facilitating infection development. Lipid nanoparticles (LNPs) serve as an effective vehicle for delivering small interfering ribonucleic acids (siRNA) that represent a method to knockdown integrin α5β1 in the lungs through nebulization, thereby potentially mitigating the severity of *S. aureus* pneumonia. The aim of this study was to harness LNP-mediated targeting to precisely knockdown integrin α5β1, thus effectively addressing *S. aureus*-induced pneumonia.

**Methods:**

C57 mice (8 week-old females) infected with *S. aureus *via an intratracheal nebulizing device were utilized for the experiments. The LNPs were synthesized via microfluidic mixing and characterized by their size, polydispersity index, and encapsulation efficiency. Continuous intratracheal nebulization was employed for consistent siRNA administration, with the pulmonary function metrics affirming biosafety. The therapeutic efficacy of LNP-encapsulated siRNAs against pneumonia was assessed through western blotting, bacterial count measurement, quantitative polymerase chain reaction, and histological analyses.

**Results:**

LNPs, which have an onion-like structure, retained integrity post-nebulization, ensuring prolonged siRNA stability and in vivo safety. Intratracheal nebulization delivery markedly alleviated the severity of *S. aureus*-induced pneumonia, as indicated by reduced bacterial load and bolstered immune response, thereby localizing the infection to the lungs and averting systemic dissemination.

**Conclusions:**

Intratracheal nebulization of LNP-encapsulated siRNAs targeting integrin α5β1 significantly diminished the *S. aureus*-mediated cellular invasion and disease progression in the lungs, presenting a viable therapeutic approach for respiratory infections.

**Supplementary Information:**

The online version contains supplementary material available at 10.1186/s12967-024-05711-9.

## Background

The lung is the most crucial organ of the human respiratory system, and its health determines the quality of life of an individual. Owing to its anatomical feature of interfacing directly with the external environment, the lung is particularly susceptible to external infections and injuries [1]. As such, diseases affecting the respiratory system represent a significant global health challenge, affecting over one billion individuals worldwide with acute or chronic conditions. *Staphylococcus aureus* (*S. aureus*) is a bacterium commonly found on human skin and mucosal surfaces, which has traditionally been considered an extracellular pathogen [2]. However, recent research has revealed its capacity to invade nonphagocytic cells such as epithelial and endothelial cells, where it can lie dormant and await opportunities for self-proliferation [3–6].

*S. aureus* infections can lead to severe illnesses, including pneumonia, endocarditis, bone and joint infections, and sepsis [7, 8]. Among these, pneumonia caused by *S. aureus* is the most common disease with a high fatality rate. The excessive use of antibiotics and the propensity of *S. aureus* for developing drug resistance necessitate the urgent development of novel anti-infection strategies. The virulence of the bacterium is significantly associated with its ability to bind directly to the extracellular matrix of the host cells.

Multiple studies have demonstrated that the primary pathway for *S. aureus* to adhere and internalize into the alveolar epithelial cells for latent survival, and therefore, escaping immune cell phagocytosis is through the binding of the fibronectin-binding protein (FnBp) on the bacterial cell surface to the extracellular matrix fibronectin (Fn), which exposed its Arg–Gly–Asp (RGD) domain for binding with the integrin α5β1 on the surface of the host cell [9–12].

Integrin α5β1 belongs to the RGD-binding integrin family, which is expressed on the surface of various cell types, including T cells, B cells, monocytes, endothelial cells, and epithelial cells [13, 14]. This receptor is involved in numerous physiological functions such as cell migration, invasion, proliferation, and aging, significantly impacting inflammatory processes. Research has established that integrin α5β1 is present in respiratory epithelial cells, and it serves as a receptor facilitating the entry of pathogens like *Staphylococcus aureus*, *Klebsiella pneumoniae*, Ebola, and SARS-CoV-2 into the respiratory system [15–18]. Therefore, the inhibition of integrin activity can weaken the bacterial adhesion and invasion of epithelial cells [19].

Ribonucleic acid (RNA) interference (RNAi) is a groundbreaking discovery in life sciences, which utilizes siRNA to specifically degrade messenger RNA (mRNA), thereby inhibiting target gene expression. Despite advancements, challenges persist in effectively delivering siRNA into target cells [20]. For instance, unprotected or unmodified siRNA is rapidly degraded by nucleases in the bloodstream, which produces a short in vivo half-life [21]. Given its typical hydrophilic polyanionic nature and approximately 13 kDa size, siRNA struggles to permeate the plasma membrane, which results in inefficient cell uptake [22]. Thus, developing more effective delivery mechanisms for siRNA to reach specific organs and cells is essential [23].

Although traditional viral vectors excel in transfection efficiency, their limitations in siRNA loading capacity and safety concerns are notable drawbacks. Conversely, nonviral vectors offer several benefits such as affordability, higher loading capacities, enhanced safety, reduced immunogenicity, and scalability for mass production [24]. Among these, the lipid nanocarriers stand out as extensively researched and are utilized as nonviral systems for nucleic acid delivery. Lipid nanoparticles (LNPs) have achieved clinical application milestones, and they are the first siRNA delivery platforms to gain approval. Their commercial formulations typically comprise DLin-MC3-DMA (MC3), 1,2-Distearoyl-sn-glycero-3-phosphocholine (DSPC), cholesterol, and DMG-PEG2000 [25, 26]. Such nanoparticles are designed to safeguard drugs from degradation, increase their stability, and facilitate the targeted release into cells [27]. The adaptability and efficiency of LNPs underscore their significance in nucleic acid therapy [28], with potential administration routes including intratracheal nebulization [29]. Despite these advancements, the clinical approval of inhaled RNA therapeutics remains pending [30], highlighting an area ripe for innovation, particularly for gene therapy and RNAi applications.

The lungs are a complex target organ because of their vast alveolar surface area. Compared with systemic delivery, localized administration offers the advantages of reduced dosage requirements and minimized side effects. Regarding RNA delivery to the lungs, the reduced presence of serum proteins on the air side comparatively lowers nuclease activity, enhancing delivery efficacy [31]. This study employed an intratracheal nebulizing device to precisely and non-invasively deliver LNP-encapsulated siRNA directly to the respiratory system [32], thereby facilitating accurate and targeted delivery of quantitative aerosols into the trachea and lungs of mice [31].

This research aimed to disrupt the progression of *Staphylococcus aureus* (*S. aureus*) pneumonia by targeting and knocking down integrin α5β1 through the intratracheal nebulization of LNP-encapsulated siRNA. After verifying the safety of the delivery method, a specialized intratracheal nebulizing device was utilized to administer LNP-encapsulated siRNA, effectively silencing the integrin α5β1 gene in the lungs of experimental animals and demonstrating the method's potential in mitigating S. aureus pneumonia progression. This approach leverages LNPs for siRNA delivery to downregulate integrin α5β1—a key receptor in bacterial lung invasion—offers a novel siRNA-based therapeutic strategy for addressing respiratory diseases such as pneumonia, asthma, chronic obstructive pulmonary disease (COPD), and viral infections.


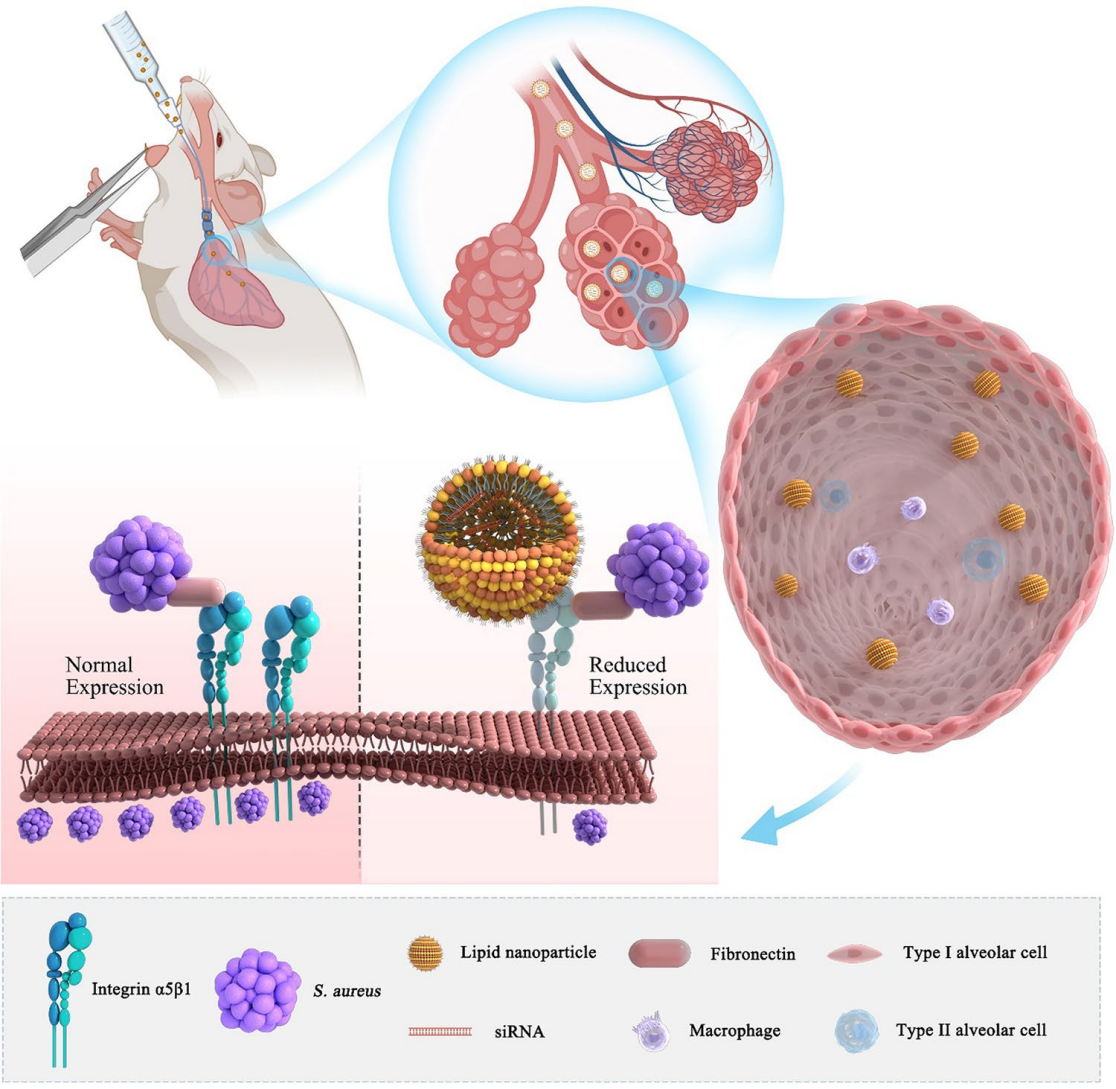


## Materials and methods

### Animals

C57 mice (8-week-old females) were acquired from Beijing Vital River Laboratory Animal Technology Co., Ltd. All experimental procedures adhered to the institutional guidelines for animal welfare and complied with the standards set forth in the Guide for the Care and Use of Laboratory Animals, receiving endorsement from the Animal Care and Use Committee of Hebei Medical University (Approval No. IACUC-Hebmu-2023063). The mice were housed in temperature-controlled, individually ventilated cages within an animal facility, subjected to a 12-h light/dark cycle, and provided with standard chow and sterile tap water.

### LNP formulation

The siRNA and Cy5 fluorescently labeled siRNA were sourced from GenePharma (Suzhou, China). The components for the LNP formulation including MC3, cholesterol, DSPC, and 1,2-dimyristoyl glycerol-rac-3-methoxy-poly(ethylene glycol)-2000 (DMG-PEG2k) were procured from A.V.T. Pharmaceutical Tech Co., Ltd. (Shanghai, China), with cholesterol additionally obtained from Nippon Seika Co. Ltd. (Osaka, Japan). The LNPs composed of MC3, cholesterol, DMG-PEG2k, DSPC, and siRNA were fabricated utilizing microfluidic mixing. In this method, an ethanol solution containing lipids was combined with an siRNA-containing buffer.

The rapid mixing process prevents the inverted micelles from aggregating, instead allowing them to be enveloped by PEG lipids, thus forming the LNP structure [33]. The siRNA was diluted in a 10-mM citrate buffer (pH 4.0), and the lipids were dissolved in ethanol. A microfluidic mixer (Harvard Apparatus, USA) facilitated the blending of these two phases at a 3:1 volume-to-volume ratio (water: oil), followed by dialysis against 0.9% w/v NaCl through a dialysis membrane with a 35-kDa molecular weight cut-off (Merck Millipore).

### LNP characterization

The efficiency of siRNA encapsulation within LNPs was quantified using the Quant-iT^™^ RiboGreen^™^ RNA Assay Kit (Invitrogen^™^ R11490) based on the concentrations of total and unencapsulated siRNA. This assay employs an ultrasensitive fluorescent stain for RNA quantification. The hydrodynamic size, polydispersity index, and zeta potential of the LNPs were assessed using a Malvern Zetasizer Nano ZS 90 (Malvern, UK). Their morphology was examined through a 200-kV field-emission transmission electron microscopy (TEM; HITACHI H-7500; Hitachi, Tokyo, Japan), with the LNPs stained with uranium peroxide acetate. For TEM analysis, 6 µL of the siRNA-LNP preparation was applied to carbon film-coated grids and allowed to dry naturally at room temperature.

### In vivo* imaging of Cy5-siRNA-loaded LNPs*

The distribution of the administered agents in organs was assessed following the pulmonary administration of both free Cy5-siRNA and LNP/Cy5-siRNA. Intratracheal spraying of these substances was facilitated by a handheld aerosolizer (MicroSprayer Aerosolizers, Yuyan Instruments Co., Ltd., Shanghai, China). Initially, mice were anesthetized and positioned supinely. Upon inserting into the trachea and reaching the carina, the sprayer needle was retracted slightly (1–2 mm) to prevent suspension splashback. A single dose of 50 µL suspension was administered. Mice were randomly assigned into groups for the quantitative pulmonary administration of both free Cy5-siRNA and Cy5-siRNA-labeled LNPs, at an siRNA dosage of 0.3 mg/kg. Various lung samples were collected at intervals of 0.5, 6, and 18 h post-administration and analyzed using the Kodak in vivo imaging system (Kodak in vivo Imaging System FX Pro; Carestream Health, USA).

### Pulmonary function test

Mice were placed in a respiratory chamber to acclimate for approximately 3 h. The respiratory function and airway responsiveness were measured using a whole-body plethysmography system designed for small animals (Whole Body Plethysmography, DSI, USA). Subsequently, anesthetized and intubated mice underwent lung function assessments utilizing a pulmonary function monitoring system (Pulmonary Function Test, DSI, USA). The process entailed setting the instrument to its default parameters before inputting related data. The mice were then positioned in the apparatus for evaluation of various pulmonary indicators through the FinePointe PFT software.

### Real-time reverse transcription-polymerase chain reaction

The total RNA was extracted using TRIZOL, and reverse transcription was conducted with the Super Script III Kit (Thermo Scientific, USA). Cytokine levels were determined via real-time polymerase chain reaction (PCR) employing SYBR Green (Vazyme, USA). Gene expression was quantified relative to the housekeeping gene (β-actin), using 2^–**△△**CT^ calculations for normalization against control values, where C_T_ denotes the threshold cycle. The primer sequences are detailed in Table 1.

### Bacterial culture

*S. aureus* (ATCC26001) was preserved at − 80 °C within the laboratory. Cryopreserved bacteria were cultured on Luria–Bertani (LB) agar plates at 37 °C for 24 h. Subsequently, a single *S. aureus* colony was inoculated into 3 mL of LB broth and incubated at 37 °C with agitation at 220 rpm overnight to multiple the bacterial population.

### siRNA

The siRNAs utilized in this study were directed against integrin α5 and integrin β1 for both sense and antisense sequences:

Integrin α5 siRNA, sense, 5′-CACCCGAAUUCUGGAGUAUTT-3′;

Antisense, 5′-AUACUCCAGAAUUCGGGUGTT-3′;

Integrin β1 siRNA, sense, 5′-GCACCAGCCCAUUUAGCUATT-3′;

Antisense, 5′-UAGCUAAAUGGGCUGGUGCTT-3′

### *Cytotoxicity *in vitro

The cytotoxicity of the LNP-encapsulated siRNA was determined by CCK-8 assay with the A549 and Raw264.7 cell line. Briefly, A549 and Raw264.7 cells were cultured in 96-well plates with an initial density of 1 × 10^4^ cells per well and cultured for 24 h, then incubated with 10 µL LNP-encapsulated siRNA at different concentrations (0, 20,50, 100, and 200 µg/ml) in complete medium for 24 h. Finally, 10 µL of CCK-8 solution was added per well and incubated for 4 h. The absorbance was measured on a microplate reader (Varioskan LUX, Thermo Scientific, MA, United States) at 450 nm.

### Western blotting

The lung tissues were harvested and lysed using radioimmunoprecipitation assay (RIPA) lysis buffer (P0013; Beyotime), supplemented with phenylmethylsulfonyl fluoride (BL507A; Biosharp) and phosphatase inhibitors (P1260; Solarbio), on ice for 30 min. The components of the lysates were denatured at 100 °C in sample buffer and subjected to SDS-PAGE before being transferred to either 0.45 mm or 0.22-mm polyvinyl difluoride (PVDF) membranes (IPVH00010; Millipore). These membranes were blocked with 5% nonfat milk for 1 h and incubated with primary antibodies overnight at 4 °C. After washing with Tris-buffered saline and Tween 20, the membranes were incubated with the appropriate secondary antibody for 1 h at room temperature, and the proteins were visualized using Western Lightning^™^ Plus ECL reagent (NEL104001EA; PerkinElmer) and detected with a Synoptics Syngene bioimaging system (R114075; Synoptics).

### Antibodies

The western blot analyses employed anti-integrin α5 (CY5979; Abways), anti-integrin β1 (CY5469; Abways), and anti-glyceraldehyde-3-phosphate dehydrogenase (GAPDH) (5174; Cell Signaling Technology) antibodies. A horseradish peroxidase-labeled goat anti-rabbit secondary antibody (ASS1009; Abgent) was used as well.

### Hematoxylin and Eosin staining

The histology of lung, liver, and kidney tissues infected with pathogens was examined using hematoxylin and eosin (H&E) staining. Mice were anesthetized with pentobarbital sodium, and either LNPs or S. aureus were administered via an intratracheal nebulizing device (25–50 µL/2 × 10^8^ CFU in 50 µL of PBS). Post-treatment, tissues were fixed in 4% polyformaldehyde, paraffin-embedded, sectioned into 5 mm-thick slices, and stained with H&E for light microscopic examination.

### Lung CFU determination

Following infection with *S. aureus* via an intratracheal nebulizing device [34], the lungs of the mice were aseptically removed and weighed. To assess intracellular viable bacterial counts, lung specimens were homogenized in RPMI-1640 (containing gentamicin) and lysed with sterile water as previously outlined. Bacterial colony-forming units (CFUs) released from lysed lung cells were quantified by culturing the lysates on Luria–Bertani (LB) agar at 37 °C for 24 h, with the bacterial burden assessed per 50 mg of lung tissue. Simultaneously, the lung tissues were processed without gentamicin, gently homogenized, washed, and then serial dilutions were cultured on LB agar plates to enumerate the “total bacteria in the lung tissue”.

### Statistical analysis

The SPSS statistical software (version 18.0) was used for the analysis, and data were expressed as mean ± standard deviation (SD). Differences between two groups were evaluated using an unpaired *t*-test, deeming a *P*-value < 0.05 as indicative of statistical significance. All experimental procedures were conducted in triplicate or more.

## Results

### *Preparation, characterization, and *ex vivo* imaging of LNPs*

**Fig. 1:**
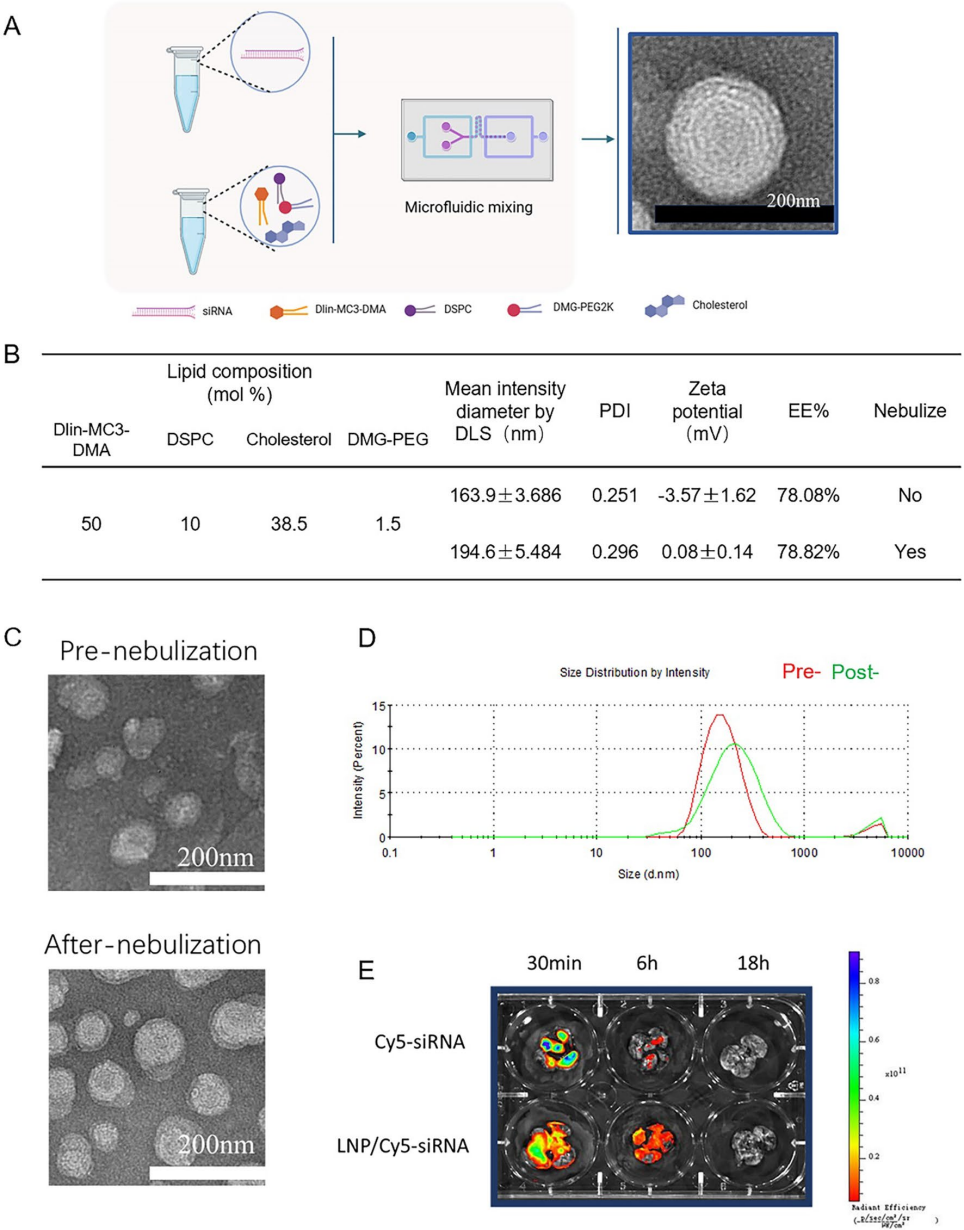
Characterization of LNPs before and after nebulization. **a** LNP preparation. **b** LNP composition. **c** TEM images of LNPs before and after nebulization. **d** LNP diameter before and after nebulization. **e** LNP/cy5-siRNA administration via nebulization to the lungs, and measurement of the fluorescence intensity (cy5-siRNA = 0.3 mg/kg)

We adopted the MC3-based LNP formulation, similar to that used in the FDA-approved RNA interference therapy Patisiran/Onpattro. The LNPs were generated using a microfluidic mixing process, achieving a final molar composition of 50:10:38.5:1.5 for Dlin-MC3-DMA:DSPC:cholesterol:DMG-PEG (Fig. 1a). The particle size measurements indicated an initial average diameter of approximately 163.9 ± 3.686 nm, which increased to about 194.6 ± 5.484 nm after nebulization (Fig. 1b, d). The polydispersity index remained below 0.3 both before and after nebulization, indicating substantial nanoparticle stability. Notably, nebulization significantly altered the zeta potential of the nanoparticles, shifting from − 3.57 ± 1.62 mV to a nearly neutral 0.08 ± 0.14 mV, thereby suggesting a potential reduction in pulmonary toxicity due to this charge change. TEM confirmed a slight increase in nanoparticle size after nebulization, which is consistent with the dynamic light scattering results (Fig. 1c, d). Moreover, the nanoparticles in the onion-like layered structure maintained a uniform spherical morphology before and after nebulization (Fig. 1a).

Cy5-labeled siRNA and LNP/Cy5-siRNA were administered to mice via an intratracheal nebulizing device at intervals of 0.5, 6, and 18 h, followed by ex vivo imaging of various tissues and organs. The initial imaging at 0.5 h revealed a notable fluorescence distribution (Fig. 1e), which indicated significant retention of LNP/Cy5-siRNA in the lungs. Although fluorescence intensity decreased by 6 h, it remained more pronounced than that observed with naked Cy5-siRNA, suggesting enhanced retention of LNP/Cy5-siRNA. By 18 h, neither treatment exhibited detectable fluorescence, pointing to the metabolic clearance of both formulations. This observation underscores the protective role of LNPs during nebulization and within the bronchi, safeguarding siRNA from degradation.

### Continuous intratracheal nebulizing delivery of LNP-encapsulated siRNA has no adverse effects on the lung function and morphology

**Fig. 2:**
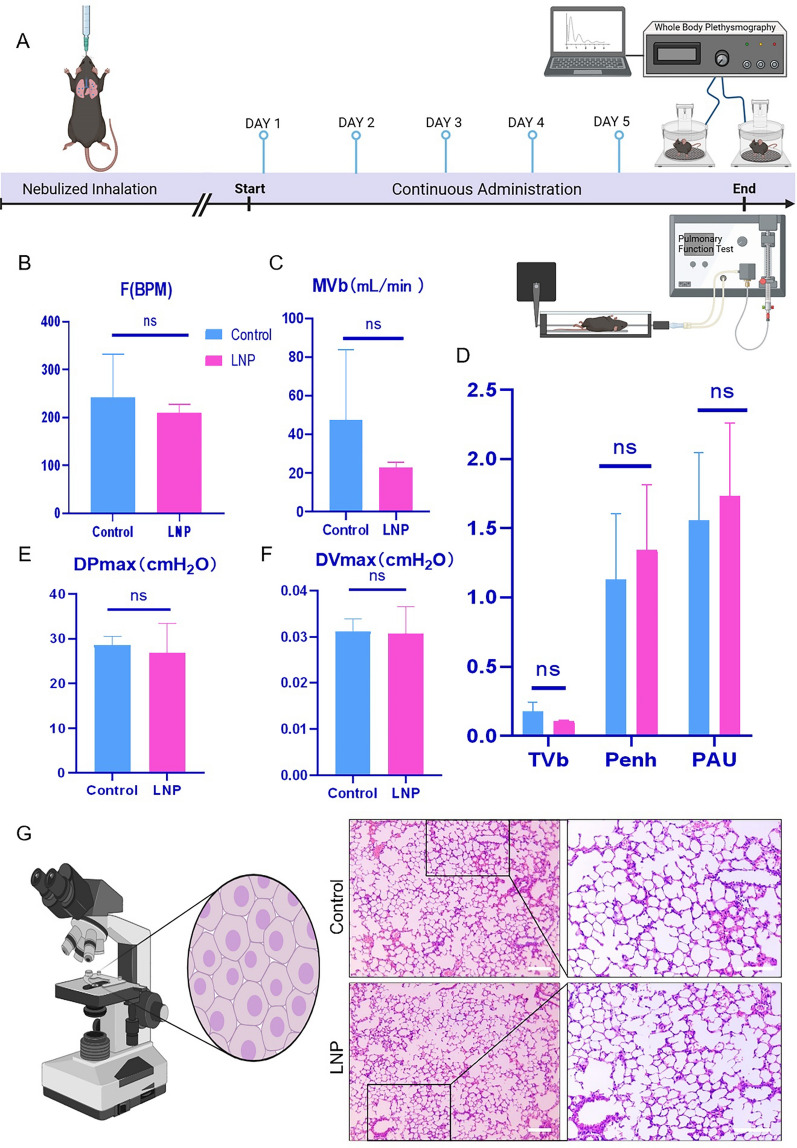
Verification of the safety of nebulized delivery of LNP-encapsulated siRNA by assessing the lung function of mice in vivo. The experiment lasted for 5 d. **A** Total of 100 µL of LNPs containing 10 µg of siRNA was delivered daily. **B**–**F** Whole-body plethysmography was performed to detect the following indices in the control and LNP-siRNA–treated groups: respiratory rate (F/BPM), ventilation per minute (MVb, mL/min), tidal volume (TVb, mL), signs of bronchoconstriction (Penh&PAU), maximum diverse pressure (DPmax, cmH_2_O), and maximum diverse volume (DVmax, cmH_2_O). **G** Lung tissues of the animals were analyzed via H&E staining. Unpaired *t*-test were used for data analysis. *ns, P* > *0.05*

Intratracheal nebulization therapy, a standard clinical approach for treating respiratory conditions, was assessed for safety regarding the continuous delivery of LNP-siRNA. Over a 5-day period, mice in both LNP-treated and control groups received 100 µL of LNPs containing 10 µg of siRNA daily (Fig. 2a). No observable differences in behavior, physical activity, eating patterns, or water intake were observed between the LNP-treated and control groups throughout the study.

The whole-body volumetry module can detect respiratory function and airway response in awake and free moving small animals. In the comparative study between the LNP treated and untreated group, multiple lung function parameters were observed), including respiratory frequency(F), minute ventilation volume (MVb), tidal volume (TVb), bronchoconstriction signs (Penh&PAU), maximum differential pressure (DPmax), and maximum differential volume (DVmax) (Fig. 2b–f). It was found that there was no statistically significant difference in any of these indices. This indicates that after five consecutive administrations, the respiratory system (including peripheral and conducting airways, chest wall, and parenchyma) is not damaged in terms of contraction and elastic stiffness.

Animal lung function test module is the ultimate test instrument for detecting all physiological indicators related to lung function. It can perform a series of group experiments on anesthetized animals, including FRC functional residual volume detection, FEV, FVC, forced vital capacity, quasi-static lung compliance, airway resistance and dynamic lung compliance, TLC lung volume, VC lung capacity and other direct physiological indicators.

**Fig. 3:**
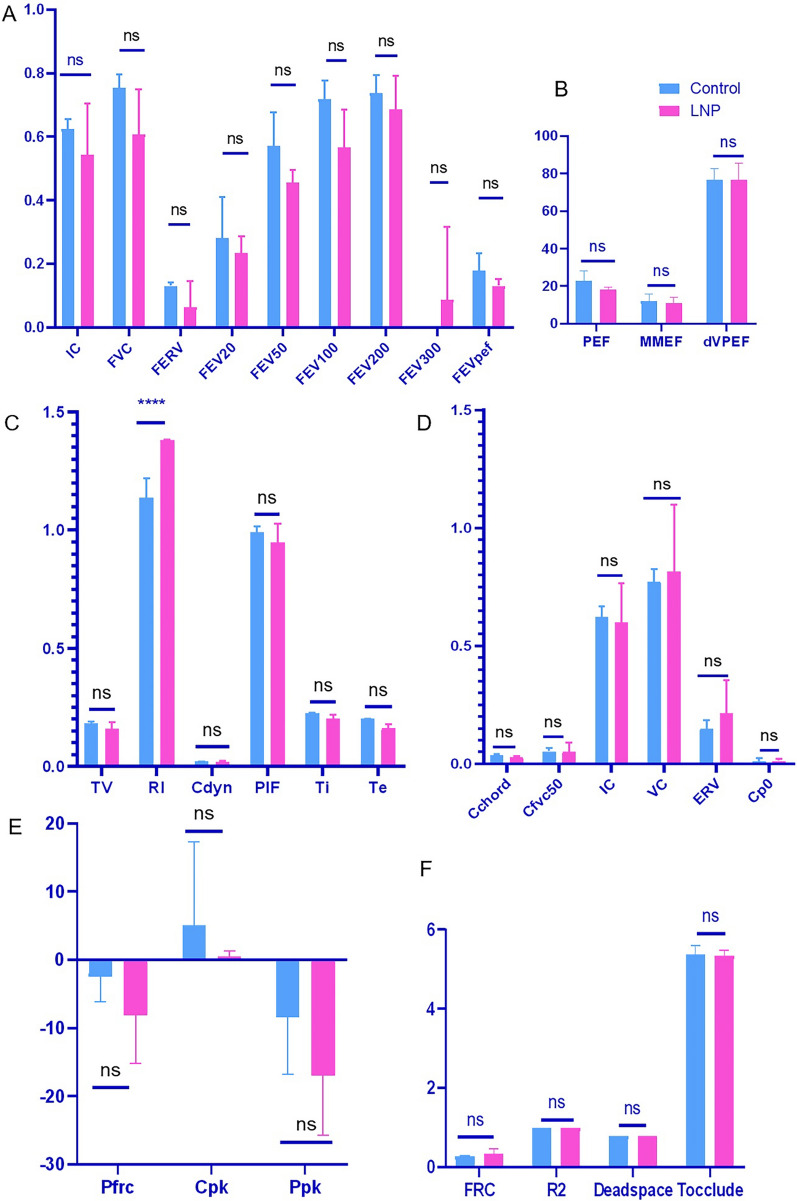
Analysis of pulmonary function test data. The respiratory function and airway responsiveness were measured using a whole-body plethysmography system designed for small animals (Whole Body Plethysmography, DSI, USA). Subsequently, anesthetized and intubated mice underwent lung function assessments utilizing a pulmonary function monitoring system (Pulmonary Function Test, DSI, USA). Pulmonary function test was operated to detect the following indices: **a**, **b** fast ventilate test (FV) consists of the following indices: inspiratory capacity (IC, mL), forced vital capacity (FVC, mL), forced expiratory reserve volume (FERV, mL), forced expiratory volume (FEV20, FEV50, FEV100, FEV200, and FEV300; mL), forced expiratory volume at the paramount expiratory flow (FEV_pef_, mL), PEF (mL), mean expiratory flow (MEF, mL/s), and residual vital capacity at the PEF (dVPEF, %). **c** Lung resistance and lung compliance tests (RC) comprising the following indices: tidal volume (TV, mL), resistance index (RI, cmH_2_O*s/mL), dynamic compliance (*C*_dyn_, mL/cmH_2_O), paramount inspiratory flow (PIF, mL/s), inspiratory time (*T*_i_, s), and expiratory time (*T*_e_, s). **d**, **e** quasistatic pressure–volume (PV) test is composed of the following components: lung compliance chord (*C*_chord_, mL/sH_2_O), lung compliance at 50% vital capacity (*C*_fvc50_, mL/sH_2_O), inspiration capacity (IC mL), vital capacity (VC, mL), expiratory repair volume (ERV, mL), compliance pressure zero (*C*_p0_, mL/cmH_2_O), pressure restore of the FRC (*P*_frc_, cmH_2_O), compliance peak (*C*_pk_, mL/cm H_2_O), and peak compliance pressure (*P*_pk_, cmH_2_O). **f** Functional residual volume test, including the following indices: functional residual capacity (FRC, mL), goodness of fit (*R*^2^), hardware volume (DeadSpace, mL), and the time the subject was occluded (*T*_occlude_, s). Unpaired *t*-test was used for data analysis. ****P < 0.001*; ns, P* > 0.05

The changes in the airways and lungs of mice after LNP treatment were evaluated in detail through mouse lung function detection. In the rapid flow test experiment (Fig. 3a, b), various indicators such as inspiratory capacity (IC), forced vital capacity (FVC, mL), forced expiratory reserve volume (FERV), forced expiratory volume (FEV20, FEV50, FEV100, FEV200, FEV300), forced expiratory volume (FEVpef), forced expiratory flow (PEF), mean expiratory flow (MMEF), and forced expiratory flow (dVPEF) reflect inspiratory capacity, ventilation function, expiratory reserve capacity, airway patency and expiratory velocity, airway patency and expiratory explosiveness, airway patency and expiratory capacity, and airway function. This indicates that in mice treated with LNP, the respiratory system is fully expanded, there is no abnormality in ventilation function and expiratory reserve capacity, and there is no airway obstruction. In the lung resistance and compliance experiment (Fig. 3c), the lung function parameters including tidal volume (TV), resistance index (RI), dynamic compliance (Cdyn), maximum inspiratory flow (PIF), inspiratory time (Ti), and expiratory time (Te) respectively indicate the depth and frequency of breathing, the level of airway resistance in the lung, the elasticity and expandability of the lung during the breathing process, inspiratory capacity and airway patency, as well as breathing rhythm and the stability of respiratory function. Considering the changes of various indicators, it can be seen that after LNP treatment, the airway resistance of the lungs increases, but there is no obvious change in lung elasticity and respiratory function. This indicates that the overall breathing process is not significantly disrupted. In the quasi-static pressure–volume experiment (Fig. 3d, e), the indicators such as lung compliance chord (Cchord), lung compliance at 50% vital capacity (Cfvc50), inspiratory capacity (IC), vital capacity (VC), expiratory reserve volume (ERV), compliance at zero pressure (Cp0), FRC pressure recovery (Pfrc), peak compliance (Cpk), and peak compliance pressure (Ppk) are related to lung compliance. The functional residual capacity (FRC) in the functional residual capacity experiment (Fig. 3f) reflects the elasticity of the lung and the patency of the airways. None of these parameters show significant differences compared to the control group mice. This indicates that the intrinsic elasticity of the lung does not change after LNP treatment.

In conclusion, through the analysis of various indicators in the lung function detection module, a comprehensive understanding of the lung function status of animals can be obtained, covering aspects such as ventilation function, airway resistance, lung compliance, and breathing rhythm. During the entire research process, observations were made on the LNP treatment group and the control group, and it was found that there were no significant differences between the two groups in terms of behavior, physical activity, diet patterns, and water intake. Thus, it can basically be determined that the intervention behavior of nebulization administration has no obvious impact on the respiratory system health of experimental animals. The alveolar structures in the LNP-treated group appeared intact with clearly defined boundaries, and the alveolar cavities were free of extraneous materials (Fig. 2g). Morphologically, no significant differences were discerned between the LNP-treated and control groups, indicating the safety of this delivery method under the experimental conditions.

Overall, the continuous delivery of LNP-encapsulated siRNA through the intratracheal nebulizing device exhibited no adverse effects on the function and morphology of the lungs under experimental conditions.

### Intratracheal nebulization delivery of LNP-encapsulated siRNAs to the lung tissue could effectively knockdown integrin α5β1

The alveolar surface features an intact epithelium comprised of type I and II alveolar cells. Type I cells are flat and extensive, covering the majority of the alveolar surface, whereas type II cells, interspersed among type I cells, contain numerous secretory granules above their nucleus [35]. The release of these granules results in the formation of a surfactant mucus layer on the alveolar surface, which serves to lower surface tension and maintain alveolar wall stability. However, this same structural configuration impedes the penetration of naked siRNA, diminishing its efficacy at the site of action. Research has demonstrated that LNP-encapsulated siRNAs can traverse the mucus layer, facilitating efficient transmucosal delivery [30].

**Fig. 4:**
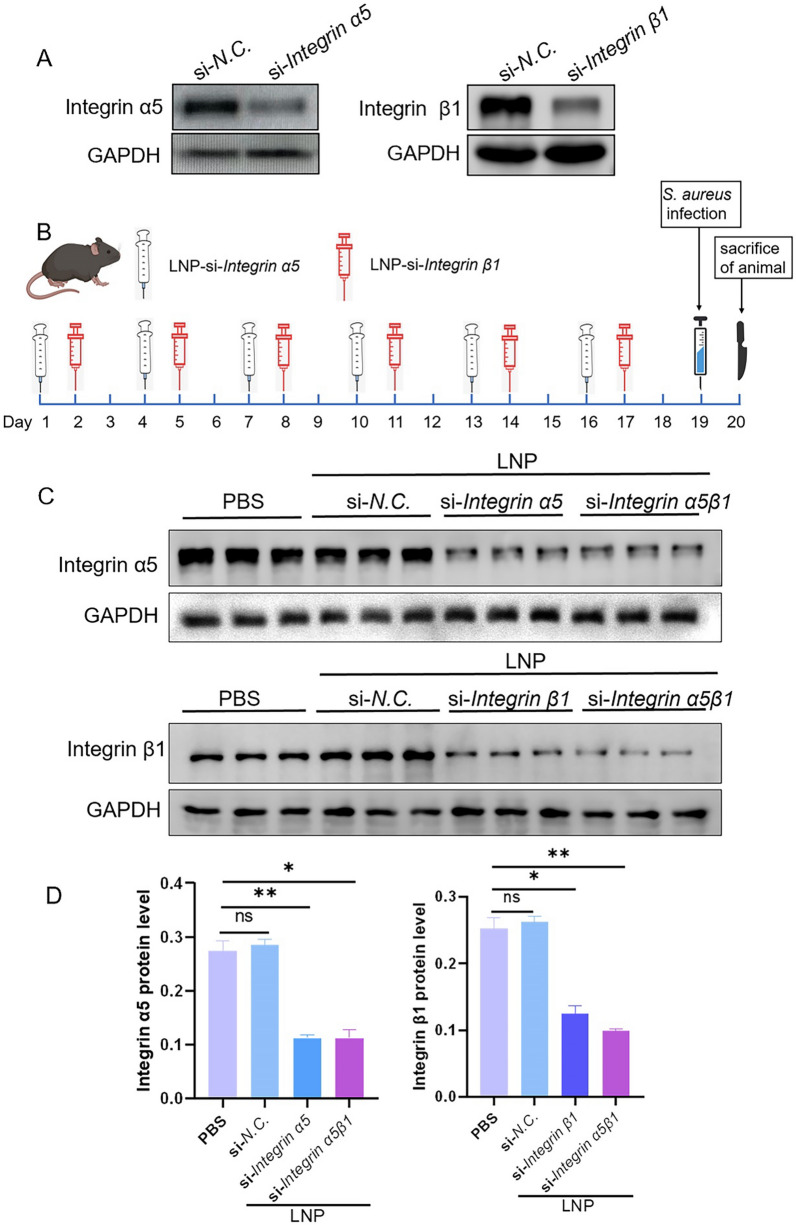
Nebulized delivery of LNP-encapsulated siRNAs to the lungs of experimental animals can effectively knockdown the target protein. **a** Knockdown effect of the siRNA sequence was verified in the Raw264.7 cell line. **b** Timeline of the experiment: the delivery was performed every 3 d in the LNP-si *integrin α5* and LNP-si *integrin β1* groups at the time intervals displayed in the figure above. LNP-si *integrin α5β1* group superimposed the delivery of the above two LNP-encapsulated siRNAs. The delivery time and quantity of the LNP-si *NC* group were consistent with those of the LNP-si *integrin α5* group. **c** Western blot analysis of the integrin α5 subunit and β1 subunit protein levels in the lung tissues of different experimental groups of mice. **d** Grayscale value analysis of the western blot results. Unpaired *t-*test was used for data analysis. *ns, P* > 0.05; *P < 0.05; **P < 0.01

Integrin α5β1 is a part of the RGD-binding integrin family, which is present on various cell surfaces, including those of respiratory epithelial cells [36–38]. This receptor has been identified as a primary entry point for numerous pathogenic microorganisms, such as *S. aureus*, into the respiratory system. Targeting this receptor with siRNA can produce anti-infective outcomes. After determining that LNP-encapsulated siRNA had no significant effect on cell proliferation in epithelial cells and macrophages, the efficacy of siRNA sequences in knocking down integrin α5β1 was initially verified in the mouse Raw264.7 cell line (Additional file 1, Fig. 4a). Subsequently, LNPs encapsulating these siRNA sequences were administered to experimental animals via an intratracheal nebulizing device (Fig. 4b).

Seventeen days post-administration, and 24 h following the establishment of the *S. aureus* infection model, lung tissues were analyzed to assess the knockdown impact. The investigation revealed that LNP-encapsulated siRNAs not only achieved effective knockdown of integrin α5β1 in vivo (Fig. 4c, d) but also underscored the high biocompatibility of LNPs and their capacity to navigate through the mucus layer, silencing target genes efficiently.

### Knockdown of integrin α5β1 could reduce intracellular viable *bacteria* and mobilize immune defenses

During the intratracheal nebulization treatment [39–41], the condition of the experimental animals was carefully monitored and documented. At the very beginning after infection with S. aureus, the animals did experience shortness of breath and decreased activity. However, with the extension of time after infection, these phenomena will gradually disappear. Throughout the study duration, no disparities were noted in the mental and motor functions between the control and LNP-treated groups. Similarly, no significant variations in body weight were detected during the administration of LNP-siRNAs (Additional file 2).

**Fig. 5:**
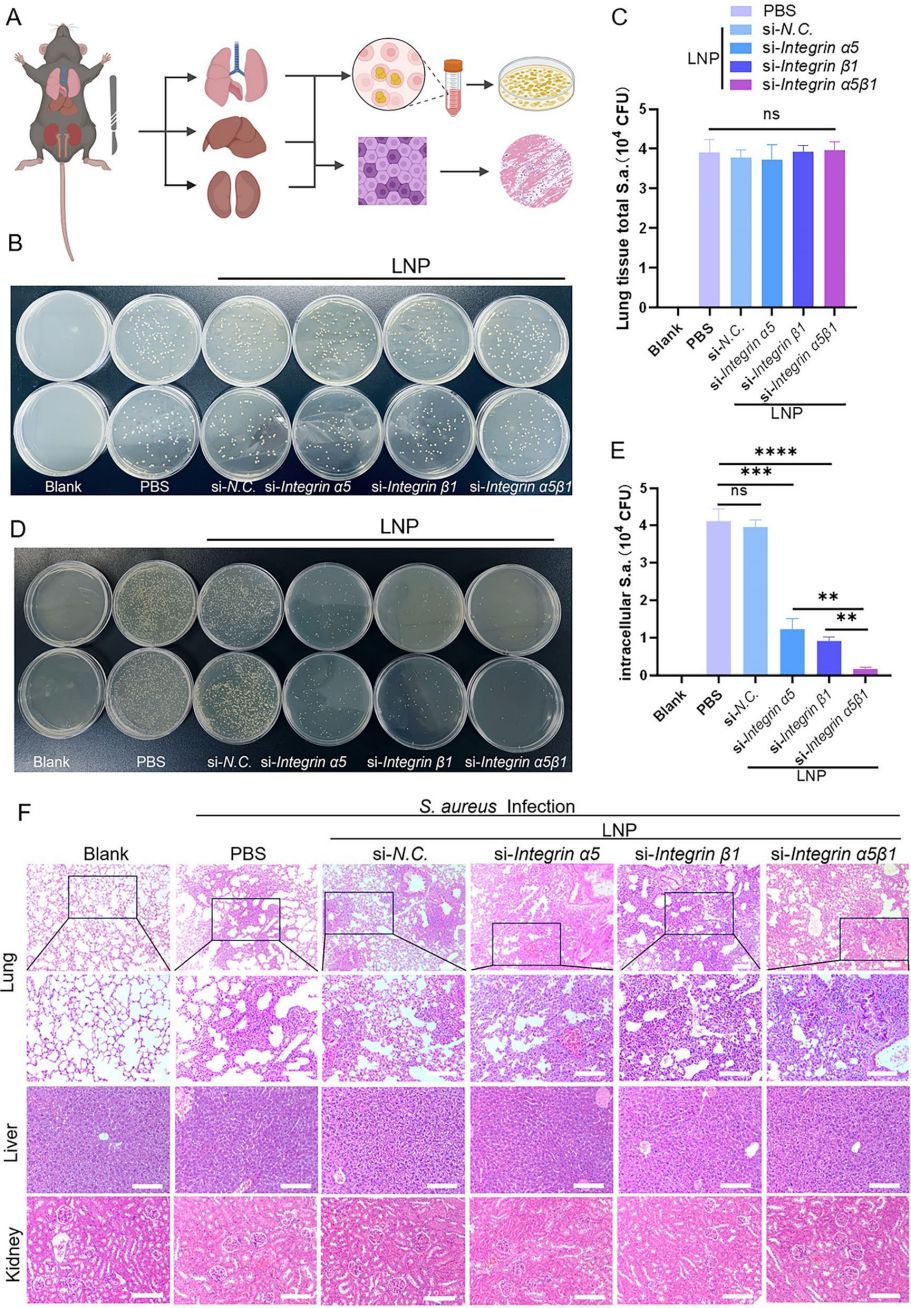
Knocking down integrin α5β1 could reduce the intracellular viable bacterial load and effectively intervene in the process of inflammation. **a** Schematic of in vivo experiment process. **b**–**e** Experimental animals were infected with *S. aureus* with the nebulization equipment (2 × 10^8^ CFU) for 24 h. Total number of intrapulmonary bacteria **b**, **c** and intracellular viable bacteria **d**, **e** were counted in the lung tissue and analyzed respectively. **f** Histological examinations of the main organs (lung, liver, and kidney)

**Fig. 6:**
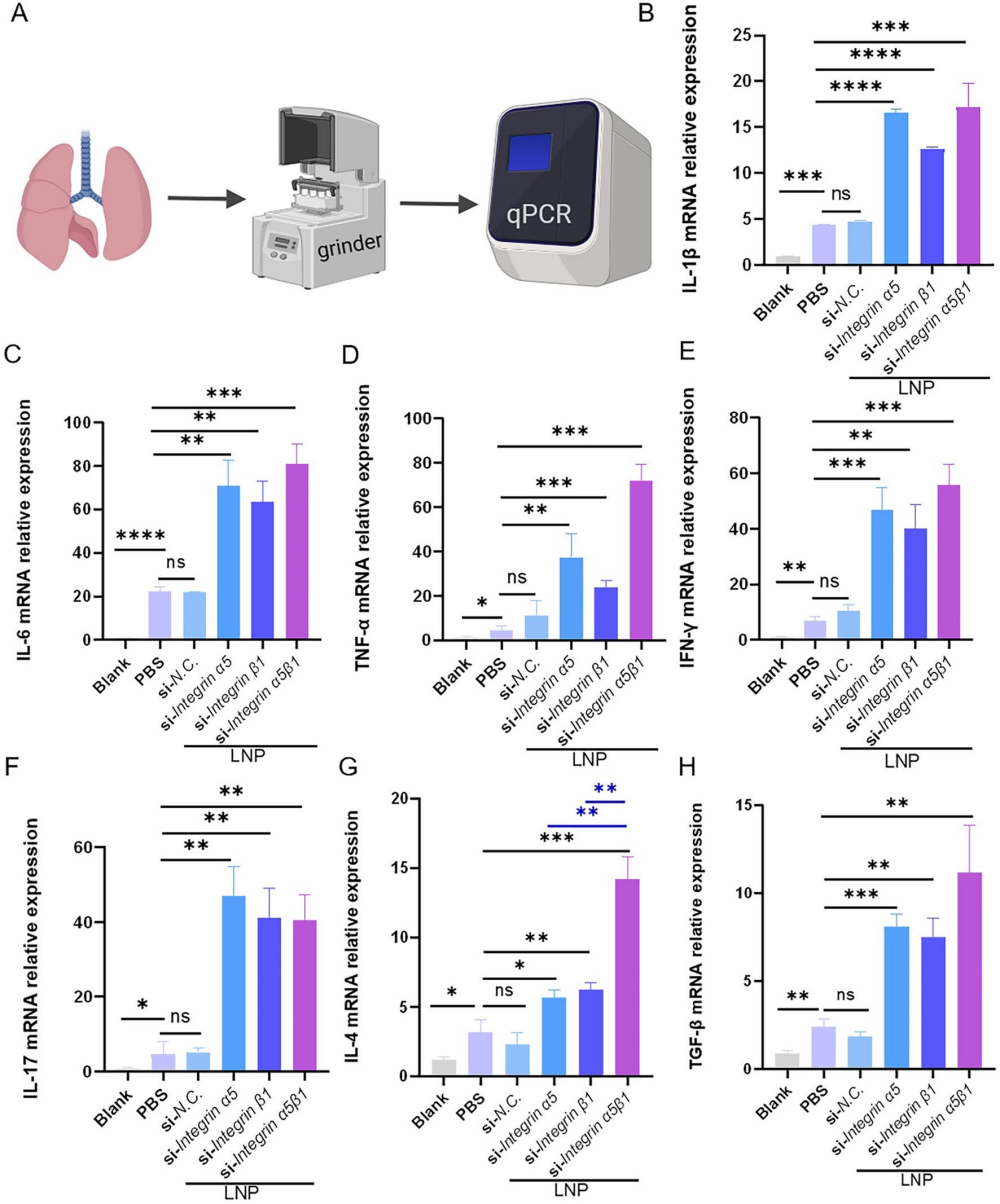
Analysis of inflammatory cytokines in the lung tissue of experimental animals in each group. **a** Schematic of RNA extraction and detection in the lung tissue. **b**–**f** Expression of proinflammatory cytokines (IL-1*β*, IL-6, TNF-*α*, IFN-*γ*, and IL-17) in each experimental group. **g**, **h** Expression of anti-inflammatory cytokines (IL-4 and TGF-*β*) in each experimental group. Ordinary one-way analysis of variance was employed for data analysis. *ns, P* > 0.05; *P < 0.05; **P < 0.01; ***P < 0.005

Following the establishment of a pneumonia infection model, the mice were euthanized 24 h after respiratory nebulization [42–44], and lung tissues from each group were harvested for analysis. This included determining total bacterial and intracellular viable bacterial counts, conducting quantitative PCR, and performing hematoxylin and eosin (H&E) staining (Figs. 5a and 6a). Upon evaluating the bacterial loads in lung tissues—weighing 50 mg each—it was discovered that despite no notable difference in total lung tissue bacterial counts, targeting integrin α5β1 subunit with LNP-siRNA significantly diminished the quantity of intracellular viable bacteria post-infection (Fig. 5b–e). Notably, simultaneous knockdown of both subunits exhibited a more pronounced resistance to bacterial invasion.

Simultaneously, compared with the PBS and LNP-si *NC* groups, after the LNP-encapsulated siRNA knockdown of integrin α5β1, proinflammatory cytokines (interleukin [IL]–1*β*, IL-6, tumor necrosis factor alpha [TNF-*α*], interferon gamma [IFN-*γ*, IL-17, IL-23, monocyte chemoattractant protein-1 [MCP-1]) (Fig. 6b–f and Additional file 3) were significantly increased in the knockdown groups, and anti-inflammatory cytokines (IL-4 and transforming growth factor beta [TGF-β]) displays the same trend as well (Fig. 6g, h). Interestingly, the expression of anti-inflammatory cytokines in the group where both subunits were knocked down was significantly higher than in the groups where only a single subunit was targeted.

The H&E-stained lung tissue sections from the control group displayed intact alveolar structures with clear boundaries and no foreign substances within the alveolar cavities. After *S. aureus* infection, the lung tissues in the PBS group exhibited significant structural disruptions, including the loss of alveolar architecture, extensive infiltration by inflammatory cells, and necrosis. Simultaneously, the LNP-siNC treated group was observed appears disintegration of inflammatory cells, the alveolar structure disintegrated and red blood cell rupture and hemolysis within vascular lumens as well as perivascular edema. Despite similar infection dosages, extracellular pathogen levels were notably higher in these groups due to fewer intracellular bacteria, resulting in a more pronounced inflammatory response in the groups treated with LNP-siRNA. *S. aureus*-induced pneumonia can escalate to bloodstream infections and, potentially, sepsis, characterized by systemic inflammation and multiorgan failure. However, the targeted delivery of LNP-siRNA and subsequent anti-inflammatory cytokine secretion localized inflammation to the lung tissues, the infection did not spread further to tissues throughout the body.

The liver tissue sections from all groups demonstrated preserved lobular architecture with clearly defined hepatocyte cords radiating around the central vein, without evidence of inflammatory cell infiltration. Similarly, the renal tissues in the LNP-siRNA-treated groups displayed no significant morphological or structural abnormalities compared to the control group (Fig. 5f). These observations further substantiate the biosafety of intratracheal nebulizing delivery of LNPs.

In summary, employing LNP-encapsulated siRNAs to target integrin α5β1 significantly reduces the load of intracellular viable bacteria in lung tissues, activates immune defenses, and recruits inflammatory cells to combat and clear the infection effectively. Importantly, the intratracheal nebulization of LNP-siRNAs did not negatively impact the morphology or function of other critical organs in the experimental animals, underscoring the therapeutic potential and safety of this approach.

## Discussion

Substantial experimental findings indicate that Staphylococcus aureus primarily invades alveolar epithelial cells via the interaction of its fibronectin-binding protein (FnBp) with the extracellular matrix fibronectin (Fn). This interaction exposes the RGD structural domain of the Fn, which facilitates its binding to integrin α5β1 on the cell surface and enabling *S. aureus* to penetrate and colonize within the cell latently [15, 19]. Given this mechanism and the broader clinical context, we hypothesized that silencing integrin α5β1 could impair the internalization and proliferation of *S. aureus*, thereby enhancing the immune response of the host, eradicating pathogens, and effectively mitigating pneumonia development. siRNAs offer specific gene expression inhibition by degrading mRNA.

Nonetheless, unprotected or unmodified siRNA faces rapid degradation by nucleases in the bloodstream and struggles to cross cell membranes due to their negative charge, which hampers cellular uptake. Leveraging our experience in developing siRNA delivery systems for cancer therapy [21, 45–48], this study utilizes a commercially available lipid nanoparticle (LNP) formulation, marking the sole marketed siRNA nanomedicine to date.

*S. aureus* infections frequently accompany various lung conditions, such as genetic disorders, cancers, infectious diseases, and chronic inflammations [49–53], which highlights the significance of direct lung-targeted drug delivery to halt disease progression. The conveyance of siRNA-loaded LNPs to non-liver targets presents notable challenges [54]. The method of drug delivery significantly influences the efficiency of carrier distribution. For pulmonary administration, direct respiratory tract delivery, like traditional aerosol inhalation, is commonly employed in treating respiratory illnesses. However, this approach suffers from limitations like low delivery efficiency and dosing inaccuracies. Intratracheal nebulization, offering enhanced dispersion and greater intra-alveolar contact, potentially surpasses oropharyngeal dripping and conventional atomizers in drug bioavailability. Moreover, intratracheal nebulization is noninvasive and can be repeatedly applied, unlike endotracheal intubation [55–57], positioning it as a promising method for siRNA drug delivery, despite its relatively unexplored status in this domain.

Recently, Massaro et al. [50] performed mRNA therapy using endotracheal delivery to target alveolar epithelial cells and fibroblasts in a pulmonary fibrosis disease model in vivo. The experimental results are encouraging for the RNA-based treatment of lung diseases.

Although this research marks significant progress in addressing S. aureus pulmonary infections, it opens avenues for further investigation. In addition to *S. aureus*, integrin α5β1 serves as a universal receptor facilitating the entry of a myriad of bacteria and viruses into alveolar cells, circumventing the phagocytosis of immune cells [58–60]. Consequently, the insights from this study advocate for a novel and more effective approach to infection prevention, offering potential targets and empirical support for counteracting pathogen intrusion.

Pulmonary diseases are often characterized by the accumulation of thick mucus within the lungs. Future investigations will aim at optimizing LNP formulations for delivery to animal models exhibiting abnormal mucus build-up. A critical aspect of enhancing the specificity of these treatments involves the incorporation of targeting ligands to precisely direct LNPs towards lung epithelial cells. Moreover, integrating antibiotics and mucolytic agents into the gene therapy-based treatments could yield superior therapeutic outcomes. Echoing the work of Li et al. [61], our forthcoming research endeavors will explore the administration of LNP-siRNA formulations via nebulizers, striving to simplify gene therapy access for patients.

Nebulized delivery systems currently face hurdles such as maintaining stability during nebulization and effectively navigating through cellular and extracellular barriers. We posit that optimizing the nebulization buffer, fine-tuning the LNP formulation ratios, crafting ionizable lipids that combine biodegradability, high delivery efficiency, straightforward chemical synthesis, and innovating noninvasive yet potent lung nebulizers could substantially enhance the pulmonary delivery of nucleic acid therapeutics.

## Conclusions

This study demonstrated that delivering LNP-encapsulated siRNA through intratracheal nebulization effectively reduces S. aureus's intracellular invasion by targeting and silencing integrin α5β1 in lung tissues. This approach shows promise in improve the host’s defense against pathogenic invasion and merits further investigation for disease intervention. The insights provided by this study may facilitate the utilization of LNPs for siRNA delivery to the lungs and promote the clinical adoption of RNA-based therapies.

## Supplementary Information

Additional file 1.

## Data Availability

The original contributions presented in the study are included in the article/Supplementary Material. Further inquiries can be directed to the corresponding authors.

## Abbreviations

Cy5: Cyanine 5
LNP: Lipid nanoparticles
siRNA: Small interfering ribonucleic acids
mRNA: Messenger RNA
PBS: Phosphate buffered saline
FRC: Functional residual capacity
PEF: Paramount expiratory flow
TEM: Transmission electron microscopy
MC3: DLin-MC3-DMA (6Z,9Z,28Z,31Z)-heptatriacont-6,9,28,31-tetraene-19-yl 4-(dimethylamino) butanoate
DMG-PEG2000: 1,2-Dimyristoyl-rac-glycero-3-methoxypolyethylene glycol-2000
DLS: Dynamic light scattering
DSPC: 1,2-Distearoyl-sn-glycero-3-phosphocholine
H&E: Hematoxylin and eosin
PCR: Polymerase chain reaction
qPCR: Quantitative PCR
LB: Luria–Bertani
FnBp: Fibronectin-binding protein
PVDF: Polyvinyl difluoride
IL: Interleukin
TNF: Tumor necrosis factor
IFN: Interferon
MCP: Monocyte chemoattractant protein
TGF: Transforming growth factor, RNAi, RNA interference
PDI: Polydispersity index
*S. aureus*: *Staphylococcus aureus*
