# Supplementary material for: Innovative nebulization delivery of lipid nanoparticle-encapsulated siRNA: a therapeutic advance for Staphylococcus aureus-induced pneumonia
Source: J Transl Med. 2024 Oct 15;22:942. doi: 10.1186/s12967-024-05711-9 (PMC11481290; doi:10.1186/s12967-024-05711-9)
Supplement: Supplementary file 1 — Additional file 1. [file 12967_2024_5711_MOESM1_ESM.pdf]

Additional Files

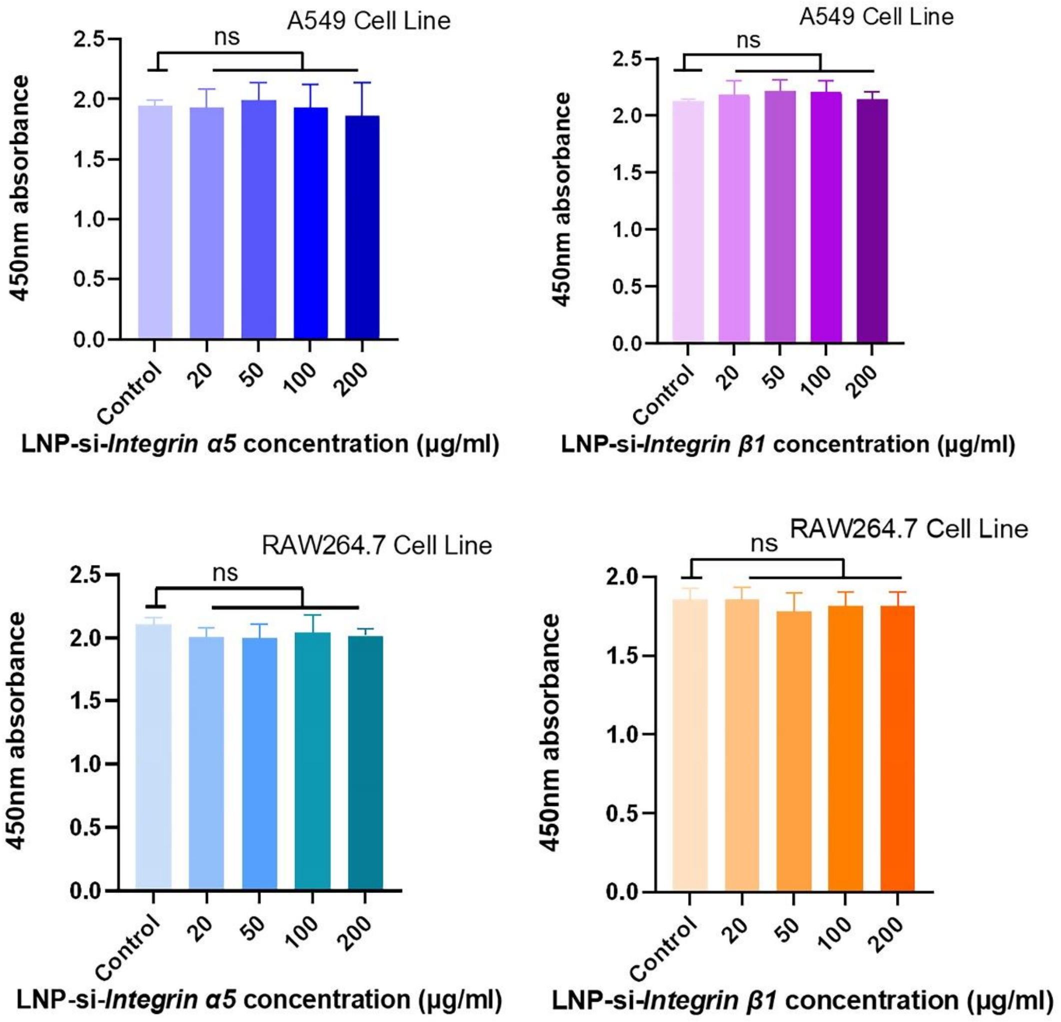

Figure S1. LNP-encapsulated siRNA had no significant effect on cell proliferation. CCK-8 assay was applied with the A549 and Raw264.7 cell line. Cells were cultured in 96-well plates with density of 1×10^4 cells per well and cultured overnight, then incubated with 10 ul LNP-encapsulated siRNA at different concentrations in complete medium for another 24 h. Finally, 10 ul of CCK-8 solution was added per well and incubated for 4 h. The absorbance was measured at 450 nm to test cell viability.

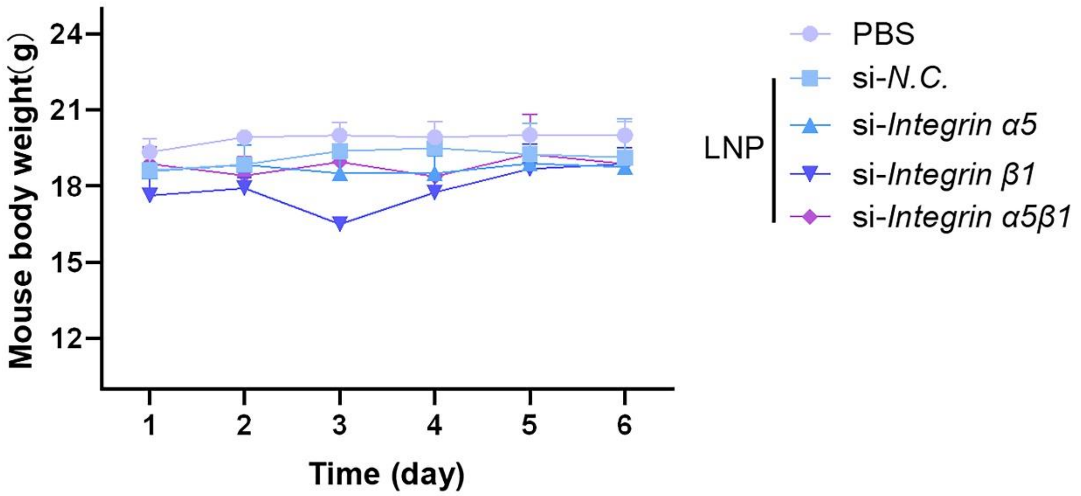

Figure S2. Continuous delivery of LNP-encapsulated siRNA had no significant effect on experimental animal body weight.

Based on the timeline in Fig. 4B, the body weight of each group of experimental animals was recorded. Data were collected for analysis prior to each delivery of the formulation. During the experimental period, there was no significant fluctuation in the body weight of the animals in each group.

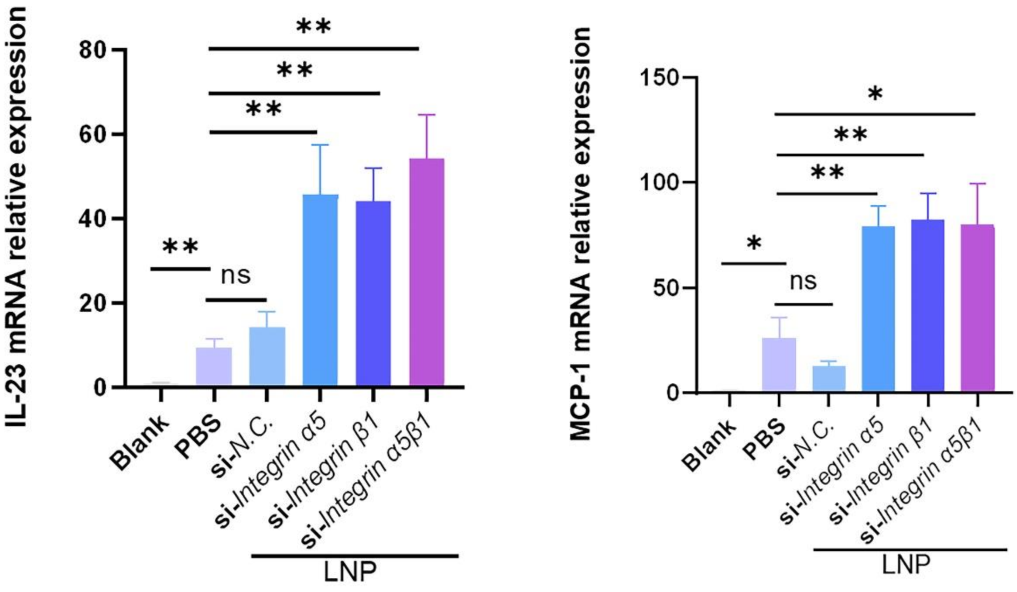

Figure S2. Supplemental analysis of cytokine levels in the lungs of experimental

groups.

After the continuous delivery of LNP-encapsulated siRNA, the lungs were collected and RNA was extracted for cytokine detection (IL-23, MCP-1). Compared with the blank group and the control groups, the levels of relevant cytokines in the LNP treatment groups were significantly increased.

TABLE 1 Primer sequences form RNA analysis by real-time PCR.

Gene Name	Primer Direction	Sequence
β-actin	Forward	TTAGGAGTGGGGGTGGCTTT
	Reverse	GTTGGTTGGAGCAAACATCCC
IL-1β	Forward	TTCCTTGTGCAAGTGTCTGAAG
	Reverse	CACTGTCAAAAGGTGGCATTT
IL-4	Forward	GCACCTTGGAAGCCCTACAG
	Reverse	GGAGATGGATGTGCCAAACG
IL-6	Forward	AAGGAGTGGCTAAGGACC
	Reverse	GTTTGCCGAGTAGATCTCAAA
IL-17	Forward	CCTCCAGAATGTGAAGGTCA
	Reverse	CTATCAGGGTCTTCATTGCG
IL-23	Forward	AATAATGTGCCCCGTATCCA
	Reverse	CTGGAGGAGTTGGCTGAGTC
MCP-1	Forward	GCTGACCCCAAGAAGGAATG
	Reverse	GAAGACCTTAGGGCAGATGCA
TGF-β	Forward	AGCCGTGGAGGGGAAATTG
	Reverse	GCGTTGATGTCCACTTGCAG
TNF-α	Forward	GCCTCTTCTCATTCCTGCTTGTGG
	Reverse	GTGGTTTGTGAGTGTGAGGGTCTG
IFN-γ	Forward	AGCAACAGCAAGGCGAAAAA
	Reverse	TGGTGGACCACTCGGATGA

TABLE 2 Routine test results of peripheral blood of mice ($\overline{x} \pm s$).

Grouping	WBC($\times 10^9$/L)	Lymph# ($\times 10^9$/L)	Mon# ($\times 10^9$/L)	Gran# ($\times 10^9$/L)
Blank	2.70±0.87	2.10±0.72	0.067±0.06	0.53±0.15
PBS	3.67±3.35 *	2.43±2.58	0.107±0.12	0.60±0.17
LNP-si *N.C.*	3.80±1.74 *	2.47±1.46	0.132 ±0.00	0.67±0.29
LNP-si *Integrin α5*	5.00±1.30 #	4.27±1.07 #	0.130±0.06	0.80±0.70
LNP-si *Integrin β1*	6.20±2.62 #	5.27±2.53 #	0.170±0.00	0.83±0.12
LNP-si *Integrin α5β1*	8.73±1.96 #	7.47±1.88 #	0.170±0.06	1.10±0.10

Grouping	Lymph%	Mon%	Gran%
Blank	77.40±5.28	3.10±1.06	19.50±4.78
PBS	73.67±0.15	2.37±1.21	23.97±13.92
LNP-si *N.C.*	74.40±0.96	2.30±0.00	23.30±0.96
LNP-si *Integrin α5*	85.70±0.87	2.53±0.32	11.77±1.00
LNP-si *Integrin β1*	83.30±4.56	2.67±1.02	14.03±3.65
LNP-si *Integrin α5β1*	85.33±2.87	2.10±0.62	12.57±2.25

Annotation : Compared to the Blank group, *$P < 0.05$; compared to the PBS group, #$P < 0.05$
